# Elevated Gab2 induces tumor growth and angiogenesis in colorectal cancer through upregulating VEGF levels

**DOI:** 10.1186/s13046-017-0524-2

**Published:** 2017-04-18

**Authors:** Chenbo Ding, Junmin Luo, Xiaobo Fan, Longmei Li, Shanshan Li, Kunming Wen, Jihong Feng, Guoqiu Wu

**Affiliations:** 1 Medical School of Southeast University, Nanjing, 210009 People’s Republic of China; 2 Department of Immunology, Zunyi Medical College, Zunyi, 563003 People’s Republic of China; 3 Department of Gastrointestinal Surgery, the Affiliated Hospital of Zunyi Medical College, Zunyi, 563003 People’s Republic of China; 4 Department of Oncology, the Affiliated Hospital of Zunyi Medical College, Zunyi, 563003 People’s Republic of China; 5 Center of Clinical Laboratory Medicine, Zhongda Hospital, Southeast University, Nanjing, 210009 People’s Republic of China

**Keywords:** Gab2, Colorectal cancer, Angiogenesis, VEGF

## Abstract

**Background:**

Grb2-associated binder 2 (Gab2) is a scaffolding protein that serves as a critical signaling amplifier downstream of tyrosine kinase receptors. Our previous study has shown that Gab2 induces epithelial-to-mesenchymal transition (EMT) and promotes metastasis in colorectal cancer (CRC). However, the role of Gab2 in CRC growth and angiogenesis remains unclear.

**Methods:**

The expression of vascular endothelial growth factor (VEGF) in different colorectal tissues was detected by immunohistochemistry and qRT-PCR to evaluate its correlation with Gab2. Lentiviral vectors bearing Gab2 gene and its small interfering RNAs were constructed and transfected into CRC cell lines. The effects of Gab2 on the cell proliferation in vitro and tumorigenesis in vivo, were examined via CCK‑8 assay, colony formation assay as well as tumorigenicity assay respectively. Moreover, to assess its potential role in tumor growth and angiogenesis, the expression of Ki67, CD34 and vascular endothelial growth factor receptor-2 (VEGFR2) were detected by immunohistochemistry in CRC cells tumors. Finally, we evaluated the impact of Gab2 on the expression of c-Myc and VEGF, and the probable effect of mechanistic targeted extracellular signal-regulated kinase (ERK) pathway in suppressing tumor growth and angiogenesis.

**Results:**

Up-regulation of Gab2 expression was found to be positively correlated with VEGF in CRC tissues. Exogenous expression of Gab2 obviously promoted, whereas silencing of Gab2 inhibited, proliferation and clone formation of human CRC cells in vitro. Of note, Gab2 enhanced tumorigenesis and tumor growth in mouse xenografts with high Ki67 expression, and led to an increased vessel density with strong CD34 and VEGFR2 activity. In addition, elevated Gab2 expression obviously up-regulated the expression of VEGF, and stimulated the activation of its downstream genes, ERK1/2 and c-Myc in CRC cells. Instead, down-regulated Gab2 expression significantly reduced the levels of VEGF, and inhibited the transduction of ERK/c-Myc pathway. Finally, we revealed that mechanistic target of mitogen-activated protein kinase (MEK) could attenuate Gab2-induced tumor growth and angiogenesis via altering VEGF and c-Myc levels.

**Conclusions:**

The results from our study suggest that Gab2 promotes intestinal tumor growth and angiogenesis through upregulation of VEGF expression mediated by the MEK/ERK/c-Myc pathway.

**Electronic supplementary material:**

The online version of this article (doi:10.1186/s13046-017-0524-2) contains supplementary material, which is available to authorized users.

## Background

Colorectal cancer (CRC) is a highly prevalent malignancy in the worldwide [1, 2]. The development of CRC occurs as a result of activation of multiple signaling pathways, which stimulate proliferation, invasion and metastasis as well as angiogenesis [3–5]. While important efforts in the prevention and early detection of CRC are ongoing, a majority of patients with metastatic colorectal cancer (mCRC) will face poor prognosis in part due to tumor angiogenesis, which is required for tumor growth and metastasis [6–9]. Therefore, the use of anti-angiogenic agent for mCRC will promise to further improve our treatment of this prevalent disease.

Angiogenesis plays a crucial role in the initiation and progression of malignant tumors, and thus is considered as an initial cancer hallmark [10, 11]. Although many studies have reported that various signaling molecules and growth factors are participated in angiogenesis, vascular endothelial growth factor (VEGF) family members are the most important pro-angiogenic factors that have been validated to date [12]. In tumors, the cancer cells are suffered from hypoxia, and vascular destabilization allows VEGF to activate dormant endothelial cells to obtain oxygen and energy [13, 14]. Then, the activation of VEGF pathway triggers a network of signaling processes that promote endothelial cell growth, migration, and survival from pre-existing vasculature [15]. In recent years, VEGF has been extensively studied in relation to CRC and corresponding hematogenous metastasis [16–18].

Grb2-associated binder 2 (Gab2), a key member of the Gab family proteins, mainly mediates phosphatidylinositol 3-kinase (PI3K) and extracellular signal-regulated kinase (ERK) signaling pathways [19]. It is well documented that Gab2 is involved in human tumorigenesis, especially in leukemia, breast and ovarian cancers [20–22]. Interestingly, overexpression of Gab2 induces endothelial cell migration in response to VEGF, whereas its depletion using siRNAs results in its reduction, suggesting that Gab2 may play an important role in tumor angiogenesis [23]. Subsequently, this assumption has gradually been explored and confirmed. For example, Gab2 induces tumor angiogenesis in NRAS-driven melanoma through the RAS/ERK signaling to upregulate hypoxia inducible factor-1a (HIF-1a) [24]. In addition, Duckworth C, et al. also found that amplification of Gab2 promotes ovarian tumor growth and angiogenesis by upregulating inhibitor of nuclear factor kappa-B kinase subunit β (IKKβ)-dependent chemokine expression [25].

We previously found that Gab2 induces epithelial-to-mesenchymal-transition (EMT) and CRC metastasis by mitogen-activated protein kinase (MEK)/ERK/matrix metalloproteinase (MMP) signaling pathway [26]. However, the relative molecular mechanisms by which Gab2 overexpression contributes to tumorigenesis and metastasis of CRC remain not well defined. In this study, we examined the roles of Gab2 in human CRC growth and angiogenesis, as well as its underlying mechanism. Our results showed that elevated Gab2 induced colorectal carcinoma growth and vascularization through upregulation of VEGF expression mediated by ERK/c-Myc signaling pathway. The current findings show for the first time that Gab2 plays a vital role in regulating CRC angiogenesis.

## Methods

### Cell culture and tissue collection

The human CRC cell lines were obtained from American Type Culture Collection (ATCC, Manassas, VA, USA). SW480 and SW620 cells were cultured in Leibovitz’s L-15 medium (GIBCO Laboratories, Grand Island, NY, USA) supplemented with 10% fetal bovine serum (FBS) (HyClone, Logan, UT, USA), 100 U/ml penicillin and 100μg/ml streptomycin. All the cells were cultured at 37 °C in a humidified air atmosphere containing 5% carbon dioxide. 30 consecutive specimens were collected form CRC patients undergoing surgical resection at Department of Gastrointestinal Surgery, Affiliated Hospital of Zunyi Medical College, between May 2015 and September 2015. None of these patients had received chemo-, radio- or immunotherapy prior to surgery. Informed consent was obtained from all patients before surgery, and our study were approved by the Ethics Committee of Affiliated Hospital of Zunyi Medical College according to the 1975 Declaration.

### Immunohistochemistry and quantification of vascular density

For the immunohistochemistry, we performed as previously described [26, 27]. The Gab2 (OriGene Technologies, USA) and VEGF (Abcam, UK) primary antibodies were used at a 1:150 dilution in the immunohistochemistry analysis. The immunostaining intensity and average percentage of positive cells were evaluated as previous reported [26]. Immunostaining reactions were evaluated by staining intensity (0, no staining; 1, light yellow; 2, buffy; and 3, brown) and the percentage of stained cells (0, ≤5%; 1, 6–25%; 2, 26–50%; 3, > 51%). Then, the staining intensity and the percentage of positive cells were multiplied to generate the immunoreactivity score for each case. Tumor vascular density was determined as previously described [28, 29]. The Ki67 (OriGene Technologies, USA), CD34 (Invitrogen, USA) and VEGFR2 (Invitrogen, USA) primary antibodies were used at a 1:75 dilution in the study of tumor growth and vascular density.

### Dicer shRNA and cell transductions

Lentiviral constructs containing Gab2 gene (LV-Gab2) and a negative control (LV-NC) were designed and provided by Cyagen Biosciences Inc. (Guangzhou, China). On the basis of the Gab2 sequence, three short hairpin RNAs were designed using the siRNA Target Finder (InvivoGene, San Diego, CA, USA). The effective Gab2-shRNA and negative control-shRNA sequence is 5′-GCACCAATTCTGAAGACAA-3′ and 5′-TTCTCCGAACGTGTCACGT-3′, respectively. Lentiviral vectors encoding short haipin RNAs were generated using GV248 vector (Genechem lnc. Shanghai, China). 70–80% confluent cells were transfected with lentivirus at multiplicity of infection (MOI) of 80 with enhanced infection solution (ENI.S) and 6μg/ml polybrene according to the manufacturer’s instructions.

### Western blot analysis

Western blot analysis was performed as previously described [27]. The following commercial antibodies were used in this study: Gab2 (OriGene Technologies, USA), VEGF and c-Myc (Abcam, UK), phospho-ERK1/2 and total ERK1/2 (Invitrogen, USA), β-actin (Immunology Consultants Laboratory, USA).

### Total RNA isolation and qRT-PCR assays

Total RNA was isolated using Trizol (Invitrogen, USA) according to the manufacturer’s instructions. The obtained RNA was first reversely transcribed into cDNA by using RT reagent Kit (TakaRa, Japan). Quantitative reverse transcription-PCR analysis was performed as previously described [26, 27]. GAPDH was used as an internal control. The sequences of primers in this section are the followings: (1) Gab2: 5′-GTGGGGGATCTGAATGTTTTTATG-3′ (forward) and 5′-GCCCCAGGGTAGAATGAAACG-3′ (reverse); (2) VEGF: 5′-CTTGCCTTGCTGCTCTACCT-3′ (forward) and 5′-CTGCATGGTGATGTTGGACT-3′ (reverse); (3) c-Myc: 5′-ACAGCAAACCTCCTCACAG-3′ (forward) and 5′-CGCAACAAGTCCTCTTCAG-3′ (reverse); (4) GAPDH: 5′-GAAGGTGAAGGTCGGAGTC-3′ (forward) and 5′-GAAGATGGTGATGGGATTTC-3′ (reverse).

### Cell counting kit‑8 assay

The Cell Counting Kit-8 (CCK-8) assay kit (Dojindo, Kumamoto, Japan) was performed as reported [28]. Transfected cells were plated in 96-well plates at a density of 4 × 10^3^/well with triplicate. At indicated time points, 10 μl CCK-8 solution was added to the cells for 2.5 h at 37 °C, and the absorbance of the cells was measured at 450 nm using an ELISA reader (BioTek, Winooski, VT, USA) according to the manufacturer’s instructions. The experiments were repeated 3 times for 5 days.

### Colony formation assays

To determine clonogenic ability, cells were trypsinized and placed in each well of a 6-well plate at a density of 4 × 10^2^ cells per well. Cells were allowed to grow for 2 weeks to form visible cell colonies, which were then fixed with methanol for 15 min and stained with 0.1% crystal violet for 20 min.

### Experiments in mice

Female BALB/C nude mice (5–6 weeks old) were purchased from CAVENS (Changzhou, China) and used for xenograft studies. 3 × 10^6^ of control and experiemental cells (SW480-NC, SW480-Gab2, SW620-si-Ctrl and SW620-Gab2si) suspended in phosphate-buffered saline (PBS) were injected subcutaneously into the right armpit of mice (six mice each group). In targeting MEK experiment, 3 × 10^6^ SW480-Gab2 cells were injected into nude mice subcutaneously. Mice were treated or not with U0126 every 5 days via tail vein for 5 weeks (25 mg/kg of U0126, six mice each group). Tumor volume was determined by external measurement according to the formula d^2^ × D/2 [30]. Mice were sacrificed after 35 days, the xenograft tumors were harvested and examined histologically. All animal experiments were approved by the Ethics Committee for Animal Experimentation of Zunyi Medical College.

### ELISA assay

Supernatants collected from CRC cells xenografts were assayed by the VEGF ELISA Kit (Invitrogen) according to the manufacturer’s instructions. Three independent experiments were performed with triplicate wells.

### Statistical analysis

All values were reported as mean ± SEM. Student’s *t*-test and one-way analysis of variance analysis were used to determine the significance of two groups and multiple groups, respectively. Correlation parameters were submitted to Pearson and non-parametric Spearman correlations. All statistical tests were two-sided and *p*-values < 0.05 were considered to be statistically significant.

## Results

### Gab2 expression is positively correlated with VEGF levels in CRC tissues

**Fig. 1:**
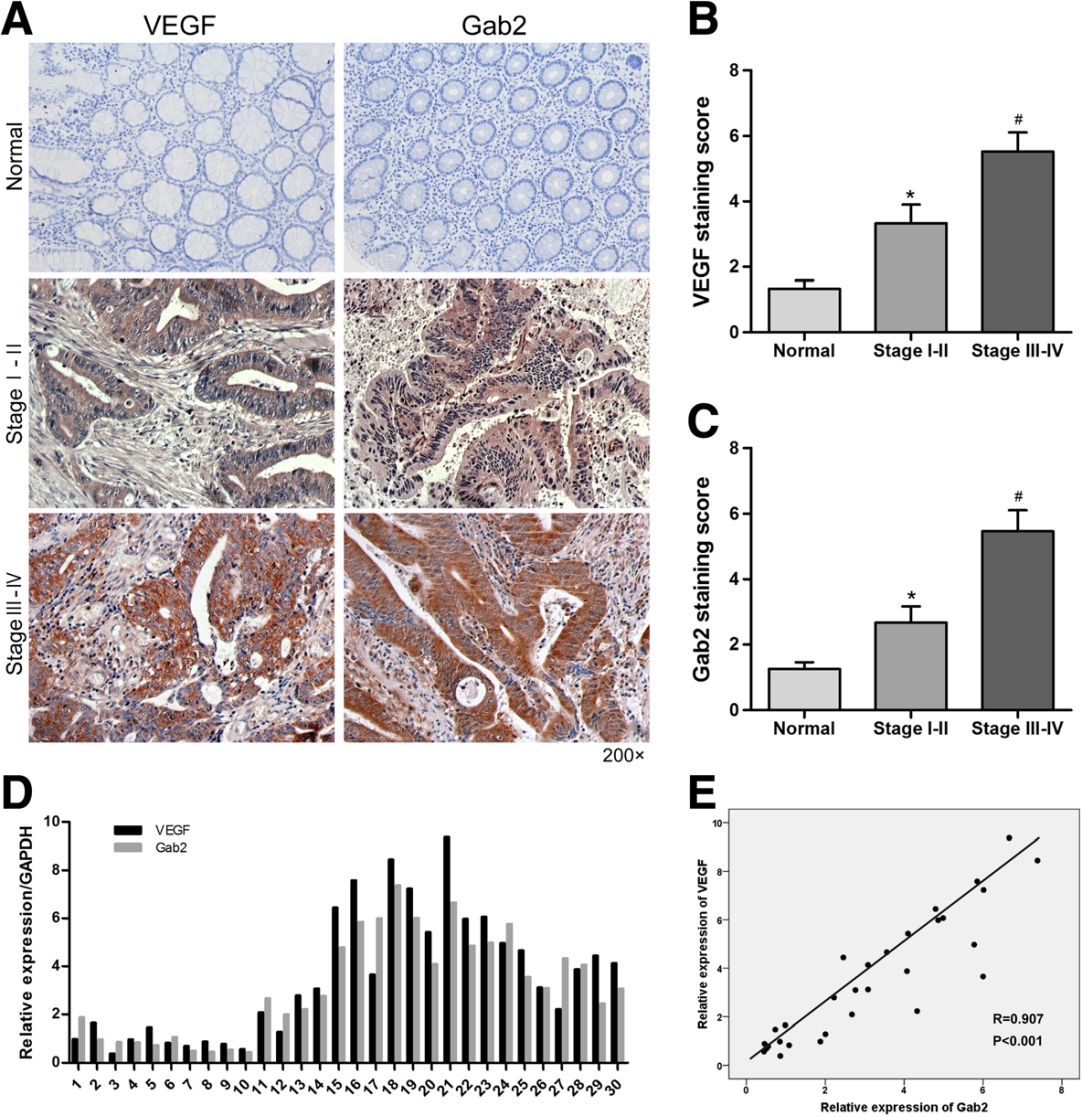
Gab2 is positively correlated with VEGF in CRC tissues. **a** Immunohistochemistry analyses of VEGF and Gab2 expression in different colorectal tissues. **b** Results of VEGF staining were evaluated by the staining scores. ^#^ *P* < 0.05 vs stage I–II or normal tissues. *P < 0.05 vs normal tissues. **c** Results of Gab2 staining were evaluated by the staining scores. ^#^ *P* < 0.05 vs stage I–II or normal tissues. *P < 0.05 vs normal tissues. **d** qRT-PCR analyses of VEGF and Gab2 expression in CRC tissues. **e** Scatter plots showing the positive linear correlation between the mRNA expression of VEGF and that of Gab2 in CRC tissues

Given the important role of VEGF in tumor growth and angiogenesis, we investigated the expression of VEGF in 15 cases of stage I–II, 15 cases of stage III–IV CRC tissues and 15 normal tissues by immunohistochemistry assay. Compared with the normal tissues, the expression of VEGF was markedly increased in stage I–II CRC tissues (Fig. 1a, b). Moreover, the levels of VEGF in stage III–IV CRC tissues were obviously higher than that of stage I–II tissues (Fig. 1a, b). In addition, the expression of Gab2 in the above different colorectal tissues also was detected, and its expression in stage I–II colorectal carcinoma was significantly higher than that of normal ones (Fig. 1a, c). Similar to VEGF, the expression of Gab2 in stage III–IV colorectal carcinoma was markedly increased than that of stage I–II tissues (Fig. 1a, c). To investigate the correlation of VEGF with Gab2 expression levels in CRC tissues, we used qRT–PCR to measure the expression levels of VEGF and Gab2 in the above 30 CRC tissues (Fig. 1d). As a result, Gab2 was found to be positively correlated with VEGF levels in CRC tissues (Fig. 1e), which indicated that Gab2 may be involved in the angiogenic process of CRC.

### Gab2 promotes CRC cell proliferation and colonigenic ability in vitro

**Fig. 2:**
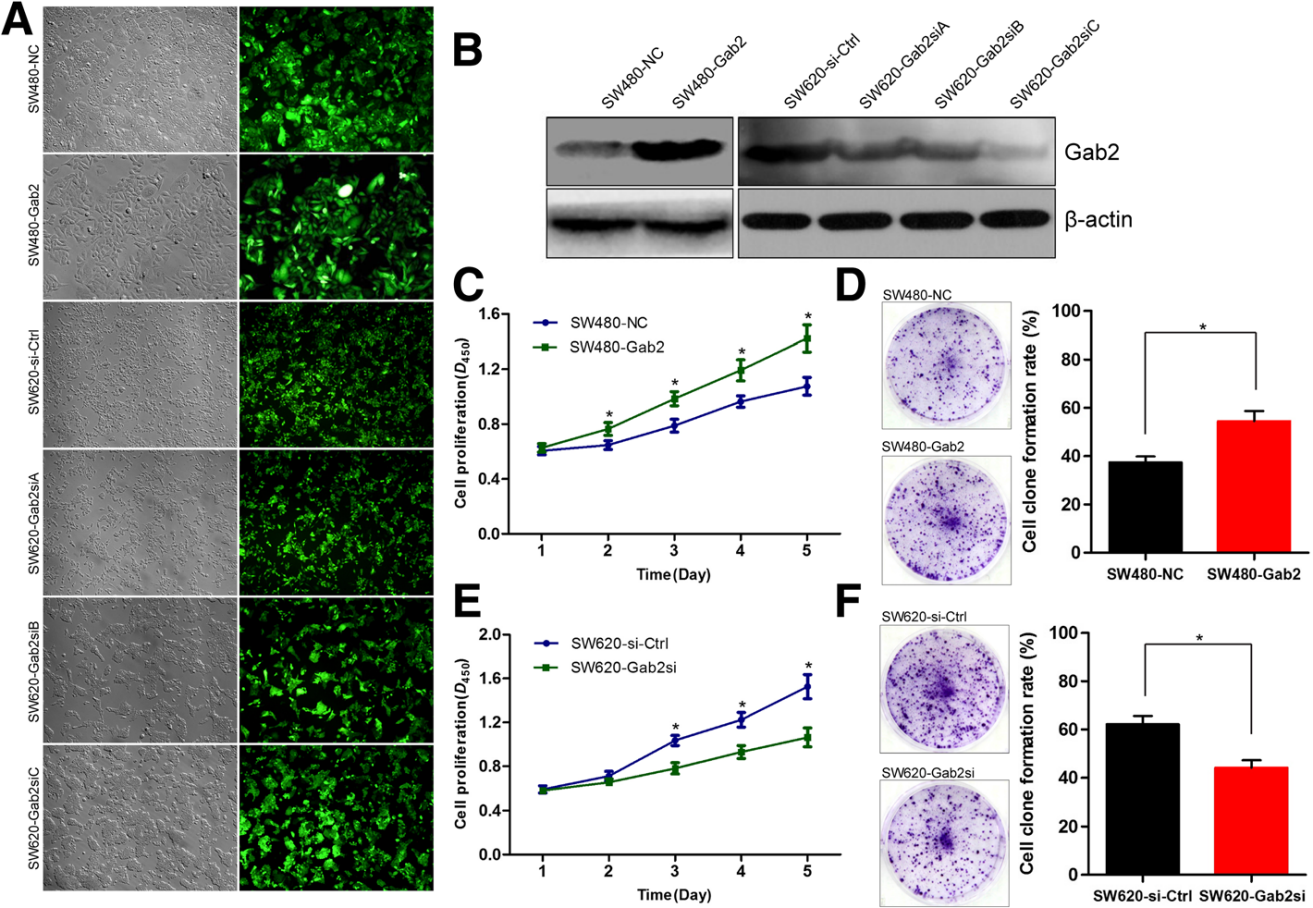
Gab2 promotes CRC cell proliferation and colonigenic ability in vitro. **a** Infected cells were examined by phase contrast microscopy (*left panel*) and fluorescent microscopy (*right panel*) and high infection efficiency was seen in these cells under fluorescent microscopy. **b** After cells were infected with LV-NC and LV-Gab2, or LV-si-Ctrl, LV-Gab2siA, -B or -C, the expression of Gab2 protein was detected by western blot analysis. **c** The proliferation ability of SW480-NC and SW480-Gab2 cells was detected by CCK-8 assay. **d** Gab2 overexpression could enhance the colony formation of SW480 cells. **e** and **f** The proliferation and colonigenic ability of SW620-si-Ctrl and SW620-Gab2si cells was assessed (two clones, **c** and **d**). Columns are the average of three independent experiments ± SEM. *P < 0.05

Our previous study has found that the different expression of Gab2 in SW480 and SW620 cells [26], which were isolated from the same CRC patient and therefore have the same genetic background [31]. We then introduced SW480 cells with recombinant lentivirus bearing Gab2 gene or a negative lentivirus and stable clones were established (SW480-Gab2 and SW480-NC, respectively) (Fig. 2a, b). Gab2-specific small interfering RNAs (siRNAs) or its corresponding control siRNA were introduced into SW620 cells. We tested three different siRNAs targeting Gab2, and selected the most effectively silenced Gab2 expression (SW620-Gab2si) for subsequent studies (Fig. 2a, b).

To determine the generality of the impact of Gab2 in regulating cell growth, we adopted the cell viability assay and colonigenic assay. As results, upregulation of Gab2 expression in SW480 cells exhibited increased cells proliferation and colonigenic ability (Fig. 2c, d). Conversely, knockdown of Gab2 expression in SW620 cells exhibited decreased cells proliferation and colonigenic ability (Fig. 2e, f). These data strongly indicated that elevated Gab2 levels in CRC cells markedly increased their proliferation and colonigenic ability.

### Gab2 induces tumorigenesis in a xenograft model

**Fig. 3:**
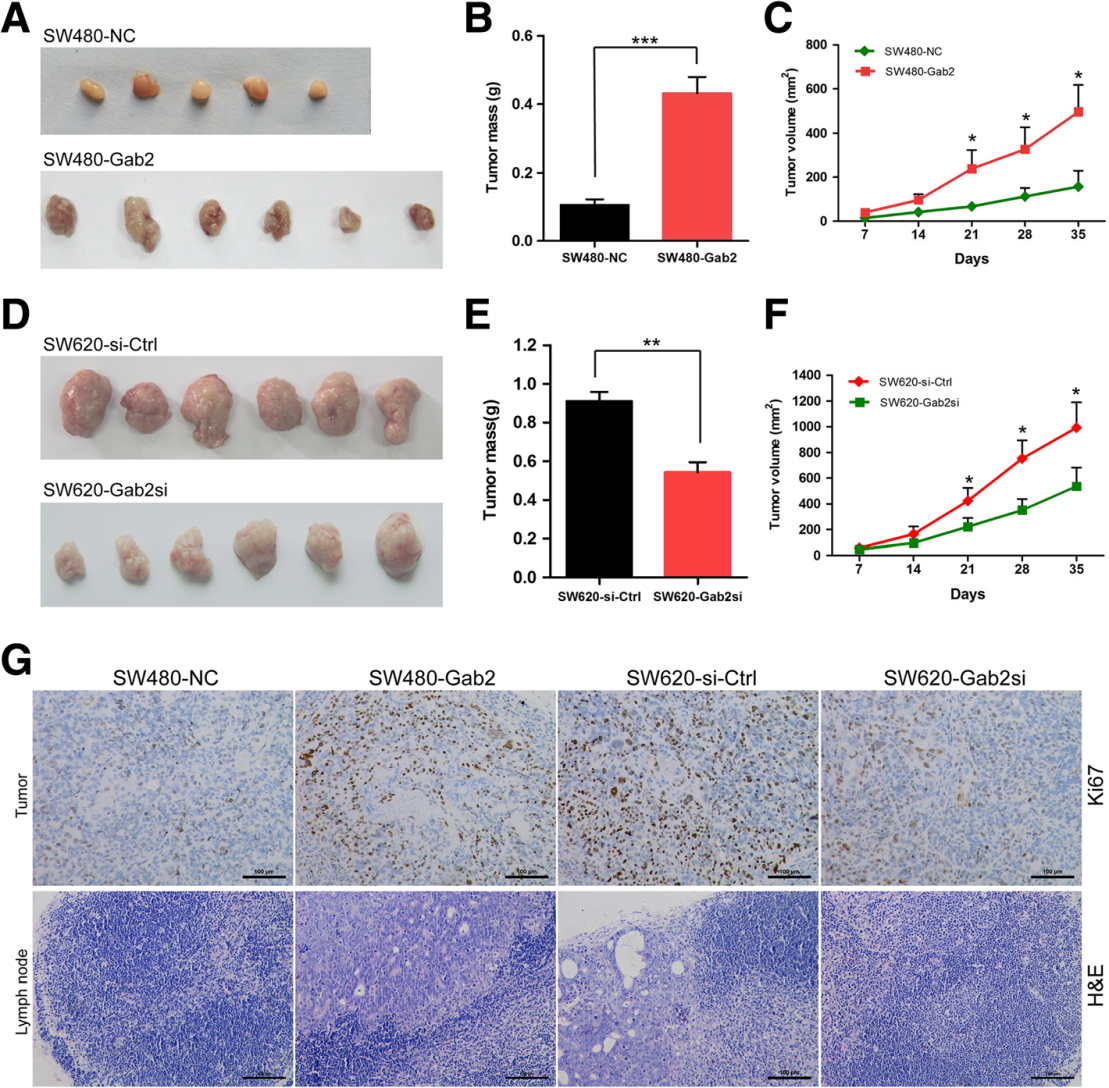
Gab2 induces tumorigenesis and cell invasion in vivo. **a**-**c** 3 × 10^6^ SW480-NC and SW480-Gab2 cells were injected subcutaneously into the *right* armpit of mice (*n* = 6 mice per group). Primary tumor growth was examined by measuring tumor volume every week. After 35 days, tumor volume and weight were analyzed. **d**-**f** 3 × 10^6^ SW620-si-Ctrl and SW620-Gab2si cells were injected into nude mice subcutaneously (three clones, **a**-**c**). **g** Xenograft tumors were analyzed for Ki67 expression using immunohistochemistry. Lymph nodes in the *right* armpit of mice were analyzed histologically through hematoxylin and eosin (H&E) staining. *P < 0.05, **P < 0.01 and ***P < 0.001

To further explore whether overexpression of Gab2 could promote tumorigenesis in vivo, the xenograft model of human CRC cells in nude mice was adopted. Cultured CRC cells (including SW480-NC, SW480-Gab2, SW620-si-Ctrl and SW620-Gab2si) were subcutaneously injected into each flank of nude mice. Tumor formation was observed and tumor weight was measured in these groups. We found that five out of six mice injected with SW480-NC cells had small xenografts (Fig. 3a). In addition, it is obviously that the tumor volume and weight from the increased Gab2 expression group (SW480-Gab2) was enhanced significantly when compared the control group (SW480-NC) (Fig. 3a, b), whereas the silencing Gab2 expression group (SW620-Gab2si) was reduced obviously when compared the control group (SW620-si-Ctrl) (Fig. 3d, e). Moreover, elevated expression of Gab2 groups (SW480-Gab2 and SW620-si-Ctrl) exhibited evidently faster growth rate when compared with the corresponding control groups (*P* < 0.05, Fig. 3c, f). Meanwhile, we found that Gab2 overexpression in implanted tumor xenografs induced CRC cells proliferation and adjacent lymphatic invasion (Fig. 3g, Additional file 1: Figure S1A). These results suggested that upregulation of Gab2 levels led to increased tumorigenesis and cell invasion in CRC.

### Gab2 promotes angiogenesis in CRC

**Fig. 4:**
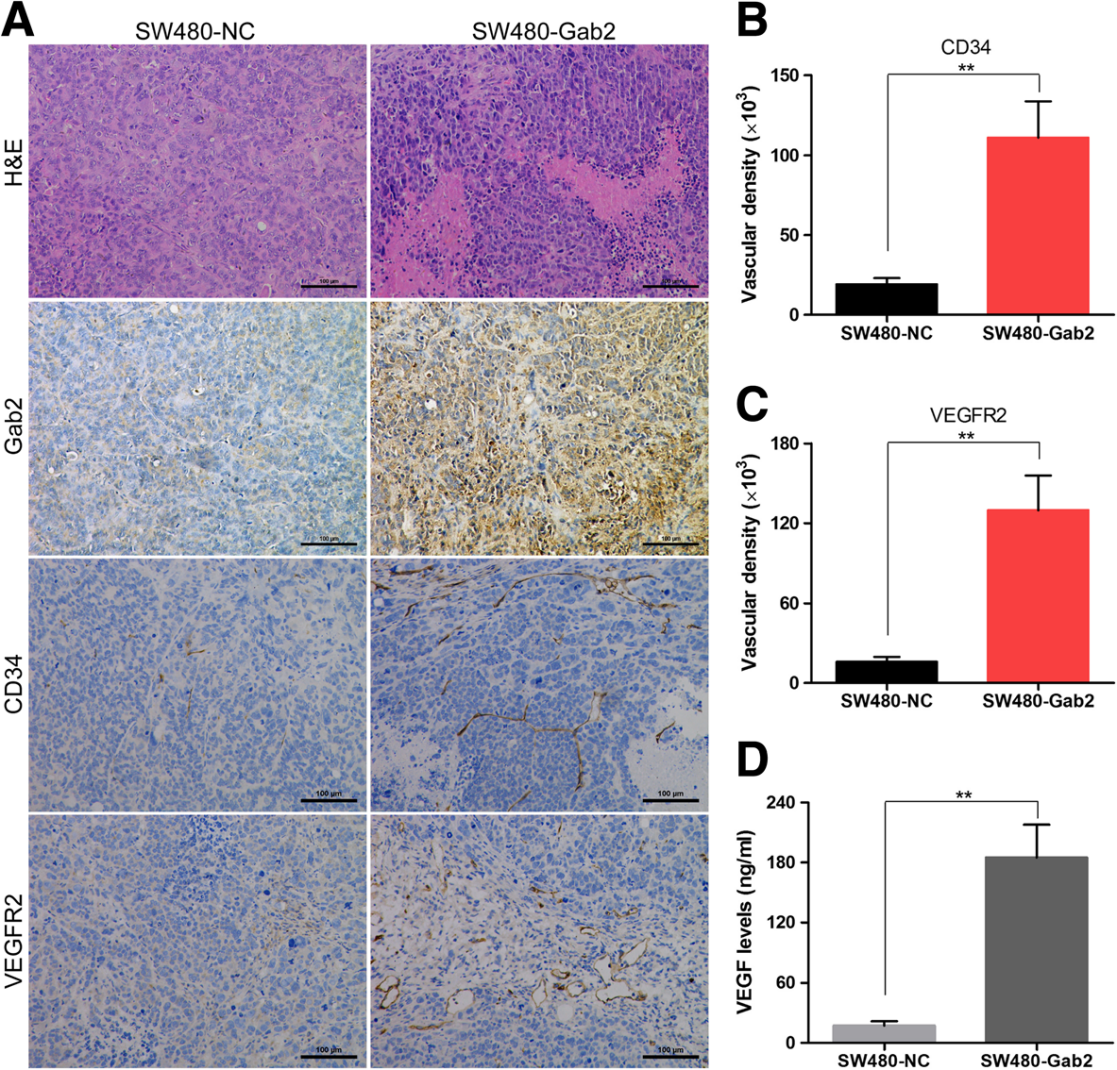
Gab2 promotes tumor angiogenesis in a xenograft model. **a** SW480-NC and SW480-Gab2 tumors were analyzed histologically using H&E staining, as well as for Gab2, CD34 and VEGFR2 using immunohistochemistry. **b** and **c** CD34 and VEGFR2-staining vasculature was quantified via measuring the vessel are as (per 200 × field, 5 fields per section) using the Image J software. **d** The levels of VEGF in SW480-NC and SW480-Gab2 tumors were detected by ELISA assay. The data are representative of at least three different experiments ± SEM. **P < 0.01

Angiogenesis plays a crucial role in the initiation, growth and metastasis of tumor. Considering Gab2 promotes the growth of CRC cells both in vitro and in vivo, we further evaluated whether elevated expression of Gab2 could induce angiogenesis in vivo. As a result, vessel numbers were significantly increased, as well as vessel length, lumina were markedly dilated in the tumors derived from SW480-Gab2 group than that in the control group (Fig. 4a). In addition, the vessels in SW480-Gab2 tumors showed strong CD34 and VEGFR2 activity (Fig. 4a, b and c). Conversely, silencing of Gab2 expression obviously decreased the CD34 staining and the amount of microvessel density (MVD) in SW620 cells groups (Additional file 1: Figure S1B and C).

VEGF as a key pro-angiogenic gene, directly stimulates endothelial cell proliferation and migration, and plays an important role in tumor angiogenesis. Therefore, we next examined VEGF levels by ELISA assay in CRC cells xenografts, and found that upregulation of Gab2 expression led to significantly higher levels of VEGF in SW480-Gab2 cells tumors than that in SW480-NC cells tumors (Fig. 4d). Instead, downregulation of Gab2 expression had the opposite effect (Additional file 1: Figure S1D). These results revealed that Gab2 might enhance CRC angiogenesis by upregulating VEGF expression.

### Gab2 enhances VEGF expression by ERK/c-Myc pathway

**Fig. 5:**
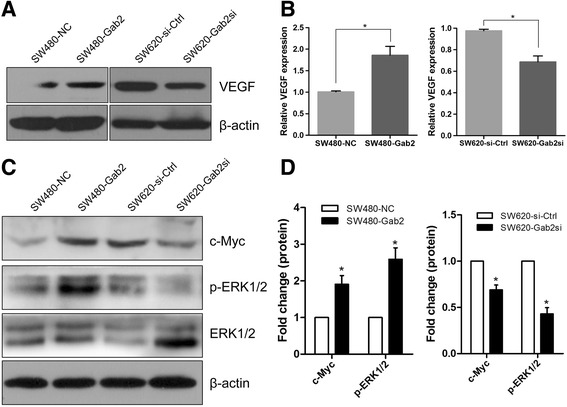
Gab2 enhances VEGF expression through ERK/c-Myc pathway. **a** Western blot analysis of VEGF expression in SW480-NC, SW480-Gab2, SW620-si-Ctrl and SW620-Gab2si cells. **b** The levels of VEGF mRNA in SW480-NC, SW480-Gab2, SW620-si-Ctrl and SW620-Gab2si cells were detected by qRT-PCR. **c** Overexpression of Gab2 enhances c-Myc expression and activates ERK1/2, whereas knockdown of Gab2 inhibits c-Myc expression and reduces ERK1/2 activation. **d** The levels of c-Myc and phosphor‑ERK1/2 were calculated. The data are representative of at least three different experiments ± SEM. *P < 0.05

As Gab2 stimulated the levels of VEGF in mouse xenografts, we asked if Gab2 expression could regulate VEGF expression in human CRC cells. The results had shown that the levels of VEGF were markedly increased in SW480-Gab2 cells and significantly decreased in SW620-Gab2si cells, compared with the corresponding control groups (Fig. 5a, b).

We previously found that Gab2 induces EMT and promotes metastasis in CRC through ERK/MMP signaling pathway [26]. c-Myc, a versatile nuclear oncogene and one of the downstream effector of the Ras/ERK pathway, is overexpressed in a variety of human tumors including CRC, and also plays an a critical role during colorectal carcinoma [32–34]. In order to further clarify the mechanism by which Gab2 promotes tumor growth and angiogenesis, and upregulates the levels of VEGF in CRC, we first looked at the expression of c-Myc in different experiment groups. Similarly to the phosphorylation of ERK1/2, upregulation of Gab2 expression in SW480 cells markedly increased the activation of c-Myc, whereas downregulation of Gab2 expression in SW620 cells obviously reduced the expression of c-Myc (Fig. 5c, d). Taken together, these results indicated that Gab2 may enhance the levels of VEGF by ERK/c-Myc signaling pathway in CRC cells.

### Mechanistic target of MEK attenuates Gab2-induced tumor growth and angiogenesis

**Fig. 6:**
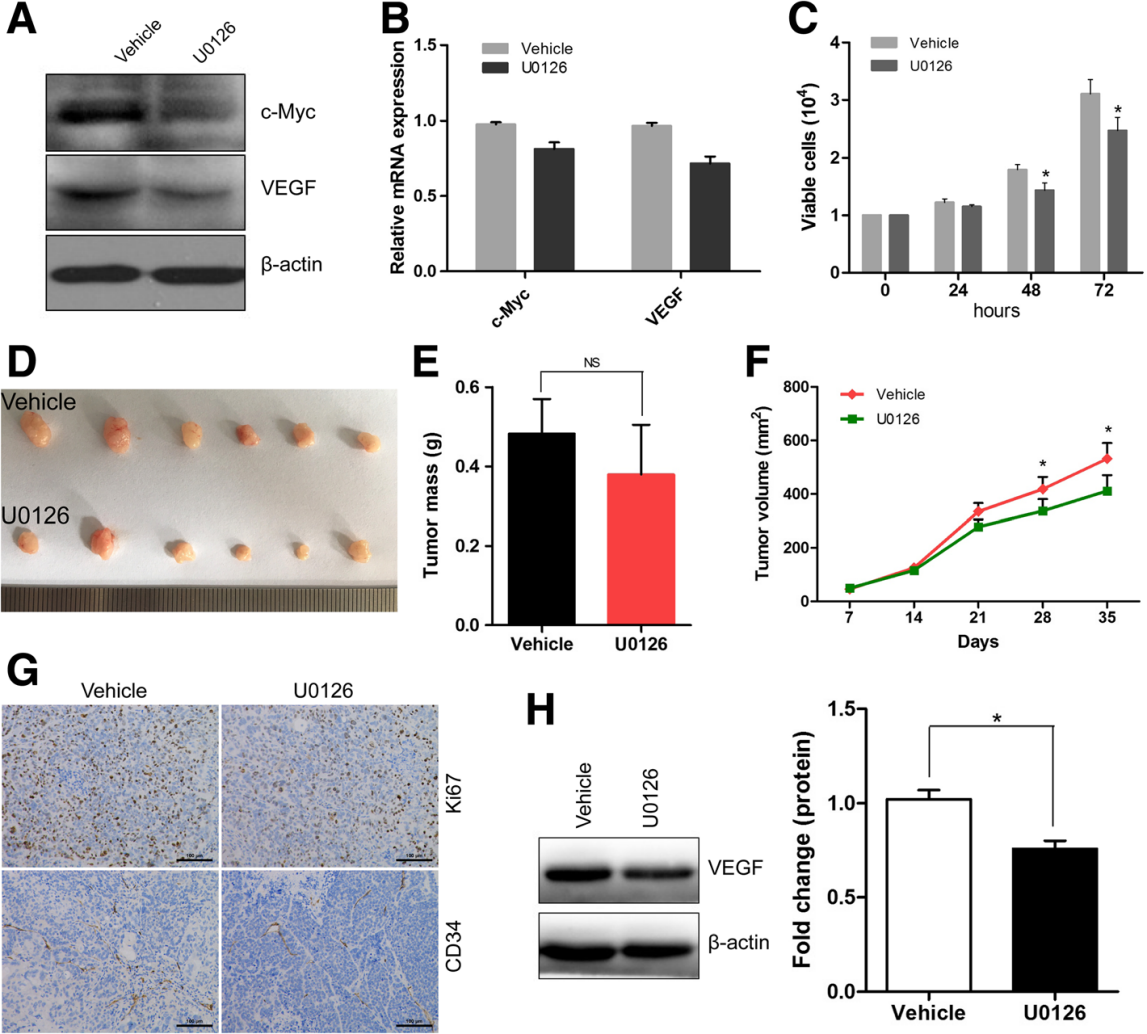
Mechanistic target of MEK attenuates Gab2-induced tumor growth and angiogenesis. **a** and **b** SW480-Gab2 cells were treated (U0126) or not (Vehicle) with 20 μM U0126 during 12 h after which proteins and mRNA were analyzed the expression of c-Myc and VEGF by western blot and qRT-PCR, respectively. **c** SW480-Gab2 cells were treated (U0126) or not (Vehicle) with 20 μM U0126, and the number of viable cells was determined 24, 48 and 72h later. **d**-**g** SW480-Gab2 cells were injected into nude mice subcutaneously. Mice were treated (U0126) or not (Vehicle) with U0126 every 5 days via tail vein for 5 weeks (*n* = 6 mice per group). Primary tumor growth was examined by measuring tumor volume every 7 days. After 35 days, tumor weight was analyzed, and xenograft tumors were analyzed for Ki67 and CD34 using immunohistochemistry. **h** Proteins were extracted from xenografts and VEGF levels were measured by western blot. The levels of VEGF were calculated. The data are representative of at least three different experiments ± SEM. NS: No statistical significance; *P < 0.05

Overexpression of Gab2 might via activation of ERK to enhance CRC growth and angiogenesis, and induce the expression of VEGF. Small molecule inhibitors for MEK, the upstream molecule of ERK, are being actively tested for CRC therapies. To find potential treatment for colorectal carcinoma with elevated Gab2 expression, we investigated whether U0126, an effective inhibitor of MEK, can suppress Gab2-induced tumor growth and angiogenesis. Gab2-overexpressing SW480-Gab2 cells were treated with DMSO (vehicle) or U0126 for 12 h and then collected for qRT-PCR and western bolt analyses. We found that MEK inhibitor could reduce the levels of c-Myc and VEGF in SW480-Gab2 cells (Fig. 6a, b). In addition, SW480-Gab2 cells treated with U0126 are resulted in the suppression of cell proliferation in a concentration dependent manner (Fig. 6c, Additional file 2: Figure S2A). To further confirm whether U0126 could inhibit Gab2-induced tumor growth and angiogenesis, we performed tumor xenograft model using BALB/C nude mice. Then, we observed that MEK inhibitor could effectively inhibit Gab2-induced tumor growth with decreased tumor volume, growth rate and Ki67 expression, but not tumor weight (Fig. 6d, e, f and g, Additional file 2: Figure S2B). Although mechanistic target of MEK only attenuates, not significantly suppresses Gab2-induced tumor angiogenesis, it works, in all (Fig. 6g, Additional file 2: Figure S2C). What’s more, U0126 could obviously alter VEGF levels in Gab2-overexpressing CRC cells tumors (Fig. 6h, Additional file 2: Figure S2D). Taken together, our results suggest that the activation of MEK/ERK/c-Myc pathway is required for Gab2-induced VEGF levels, tumor growth and angiogenesis.

## Discussion

The mortality from CRC has decreased slightly over the past decade, but approximately 20% patients will develop into metastatic disease [6, 35]. When colorectal carcinoma lesions have spread into blood or other organs, patients will have very limited options for target agents and conventional chemotherapy. According to the European and US guidelines, several targeting-therapies have been recommended for the treatment of mCRC [36]. However, the most patients have not a good response to these treatments and then lead to relapse with chemo-resistance. Therefore, traditional chemotherapy need to be improved, and novel drug targets for personalized precision medicine may be the most effective strategy for each patient based on genetic characterization of the cancer [37]. We previously performed studies have identified that Gab2 is amplified in approximately one-half of CRC tissues, and can serve as a novel oncogene for CRC metastasis [26, 27]. In the present study, we provided additional evidence showing the involvement of Gab2 in regulation of tumor angiogenesis. We found that overexpression of Gab2 in CRC cells induced tumor growth and angiogenesis through upregulating the levels of VEGF mediated by ERK/c-Myc pathway.

It has been reported that Gab2 expression is required for human tumorigenesis and tumor growth by increasing cell proliferation and independent growth [19]. Interestingly, a previous study has underscored the non-redundant and essential roles of Gab2 in VEGF-mediated signaling, and suggested major contributions of the protein during in vivo angiogenesis [23]. VEGF not simply promotes tumor angiogenesis by stimulating endothelial cell proliferation and migration, altering blood vessel permeability, but controls the functional and morphological form of these vessels, which also contribute to CRC growth and progression [35, 38]. In this study, we demonstrated that Gab2 expression was positively correlated with the levels of VEGF in CRC tissues. In addition, elevated Gab2 promoted cell proliferation and clone formation in CRC, whereas silencing of Gab2 had the opposite effects. Notably, overexpression of Gab2 in CRC cells induced tumor growth and angiogenesis in mouse xenografts through enhancing VEGF expression. Consistent with our findings, Gab2 was reported to be an inducer of tumor angiogenesis essential for melanoma and ovarian cancer [24, 25]. Our results provided new evidence supporting the involvement of Gab2 in driving tumor angiogenesis.

Our prior study showed that Gab2 facilitates EMT and metastasis in CRC, and these functions are mainly dependent on the activation of MEK/ERK signaling [26]. In order to further clarify the underlying mechanism by which Gab2 promotes tumor growth and angiogenesis by the ERK pathway, we first looked at the expression of c-Myc, a versatile pro-oncogene in CRC [34]. Similarly to ERK1/2 phosphorylation, increased expression of c-Myc was been found in Gab2-upregulated CRC cells, whereas decreased levels in Gab2-downregulated ones. In addition, Gab2-overexpressing SW480-Gab2 cells treated with the inhibitor of MEK were resulted in the suppression of Gab2-enhanced cell proliferation in vitro, and the levels of VEGF and c-Myc. Although mechanistic target of MEK has an imperfect role in reducing Gab2-induced tumor growth and angiogenesis, in all, it works. The above findings implied that Gab2-induced tumor growth and angiogenesis in CRC may beyond the control of MEK/ERK signaling.

It has been widely recognized that endothelial cell growth factor receptor (EGFR) has a potent effect on tumor associated angiogenesis, and combined treatment with EGFR and VEGF signaling inhibitors has at least additive antitumor activity [35]. Meanwhile, Gab2 is a scaffolding protein acting downstream of both EGFR and VEGF, which mediates several intracellular pathways, such as AKT, ERK and signal transducer and activator of transcription-3 (STAT3) signaling [19, 23]. Of note, a recent study suggested that VEGF-mediated CRC cell survival is dependent on AKT and ERK1/2 signaling via an intracellular mechanism, not paracrine or autocrine model [39]. According to these findings, we speculate that the plausible reasons for imperfect effect of MEK inhibitor in suppressing of Gab2-induced VEGF expression and tumor angiogenesis are listed as following: 1) Although Gab2 expression does not marked effect the phosphorylation of AKT in some CRC cells, the activation of AKT and other intracellular signaling may also play important roles in Gab2-induced tumor angiogenesis; 2) Mechanistic target of MEK may be only impact Gab2-induced VEGF expression via the intracellular mechanism, but not the paracrine or autocrine way in tumor microenvironment; 3) EGFR and other growth factors are also involved in Gab2-enhanced VEGF levels and tumor angiogenesis through an independent model of Gab2 status.

## Conclusions

Taken together, our data demonstrated that overexpression of Gab2 contributed to MEK/ERK/c-Myc signaling–enhanced tumor growth and angiogenesis of human colorectal carcinoma through upregulating the levels of VEGF. Whereas further studies are needed to pinpoint the molecular mechanisms by which Gab2-dependent ERK phosphorylation induces VEGF expression. The present study suggested that the Gab2/ERK/VEGF pathway might be an attractive target for therapeutic intervention against the growth and hematogenous metastases of CRC.

## Additional files

Additional file 1: Figure S1. Silenced Gab2 inhibits tumor growth and angiogenesis. A Ki67 staining-positive cells in SW480-NC, SW480-Gab2, SW620-si-Ctrl and SW620-Gab2si tumors were quantified. B-D SW620-si-Ctrl and SW620-Gab2si tumors were analyzed for CD34 expression using immunohistochemistry. CD34-staining vasculature was quantified via measuring the vessel are as (per 200 × field, 5 fields per section) using the Image J software. The levels of VEGF in tumors were detected by ELISA assay. The data are representative of at least three different experiments ± SEM *P < 0.05. (TIF 10744 kb)

Additional file 2: Figure S2. U0126 attenuates Gab2-induced CRC cells growth and tumor angiogenesis. A The proliferation rate of SW480-Gab2 cells were analyzed by CCK-8 assay in different U0126 concentration. B-D SW480-Gab2 cells were injected into nude mice subcutaneously in the absence (Vehicle), or presence of U0126 (*n* = 6 mice per group). Ki67 staining-positive cells and microvessel density (MVD) in tumors were quantified (per 200 × field, 5 fields per section). And the levels of VEGF protein in tumors were detected by ELISA assay. The data are representative of at least three different experiments ± SEM. NS: No statistical significance; *P < 0.05. (TIF 7542 kb)
