# Supplementary figures and images for: Elevated Gab2 induces tumor growth and angiogenesis in colorectal cancer through upregulating VEGF levels
Source: J Exp Clin Cancer Res. 2017 Apr 18;36:56. doi: 10.1186/s13046-017-0524-2 (PMC5395829; doi:10.1186/s13046-017-0524-2)

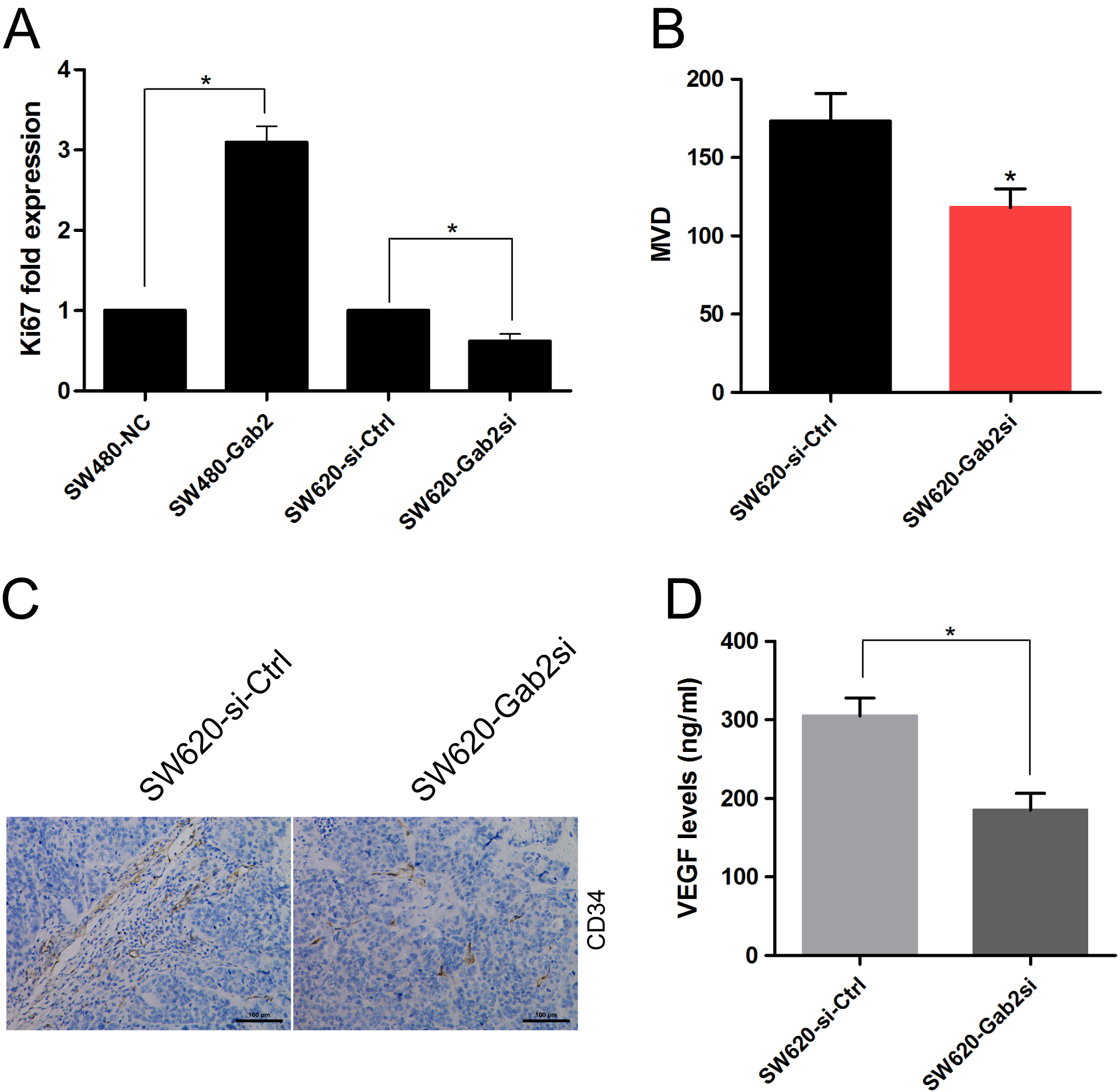

Supplement: Supplementary file 1 — Silenced Gab2 inhibits tumor growth and angiogenesis. A Ki67 staining-positive cells in SW480-NC, SW480-Gab2, SW620-si-Ctrl and SW620-Gab2si tumors were quantified. B-D SW620-si-Ctrl and SW620-Gab2si tumors were analyzed for CD34 expression using immunohistochemistry. CD34-staining vasculature was quantified via measuring the vessel are as (per 200 × field, 5 fields per section) using the Image J software. The levels of VEGF in tumors were detected by ELISA assay. The data are representative of at least three different experiments ± SEM *P < 0.05. (TIF 10744 kb) [file 13046_2017_524_MOESM1_ESM.tif]

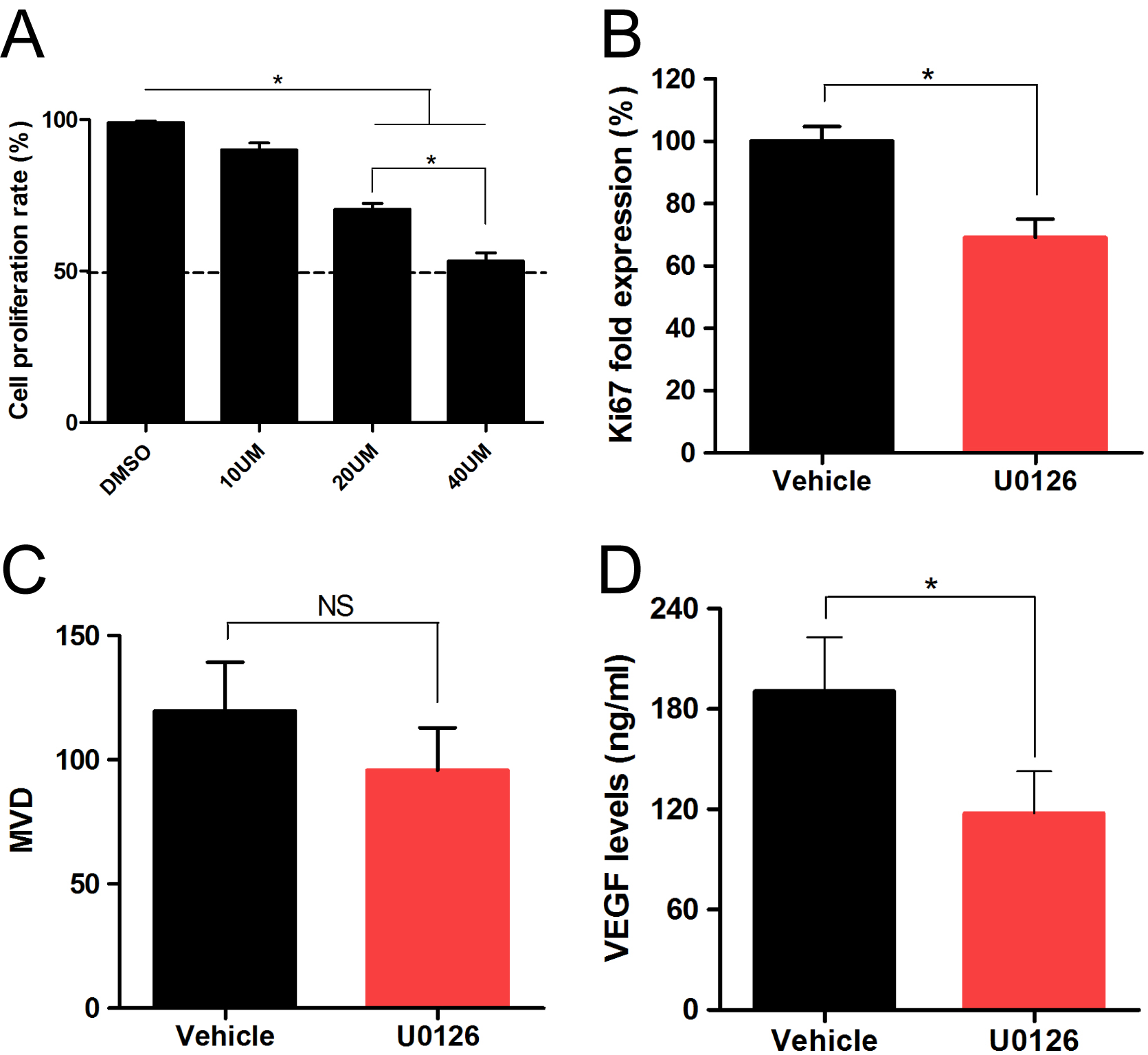

Supplement: Supplementary file 2 — U0126 attenuates Gab2-induced CRC cells growth and tumor angiogenesis. A The proliferation rate of SW480-Gab2 cells were analyzed by CCK-8 assay in different U0126 concentration. B-D SW480-Gab2 cells were injected into nude mice subcutaneously in the absence (Vehicle), or presence of U0126 (n = 6 mice per group). Ki67 staining-positive cells and microvessel density (MVD) in tumors were quantified (per 200 × field, 5 fields per section). And the levels of VEGF protein in tumors were detected by ELISA assay. The data are representative of at least three different experiments ± SEM. NS: No statistical significance; *P < 0.05. (TIF 7542 kb) [file 13046_2017_524_MOESM2_ESM.tif]
